# Additively Manufactured Parts from AA2011-T6 Large-Diameter Feedstocks Using Friction Stir Deposition

**DOI:** 10.3390/ma16144904

**Published:** 2023-07-09

**Authors:** Naser A. Alsaleh, Mohamed M. El-Sayed Seleman, Ahmed M. M. Hassan, Mohamed M. Z. Ahmed, Sabbah Ataya, Fahamsyah H. Latief, Akrum Abdul-Latif, Mohamed I. A. Habba

**Affiliations:** 1 Department of Mechanical Engineering, College of Engineering, Imam Mohammad Ibn Saud Islamic University, Riyadh 11432, Saudi Arabia; naalsaleh@imamu.edu.sa (N.A.A.); smataya@imamu.edu.sa (S.A.); 2 Department of Metallurgical and Materials Engineering, Faculty of Petroleum and Mining Engineering, Suez University, Suez 43512, Egypt; mohamed.elnagar@suezuniv.edu.eg; 3 Mechanical Department, Faculty of Technology and Education, Suez University, Suez 43518, Egypt; ahmed.mostafa@ind.suezuni.edu.eg (A.M.M.H.); mohamed.atia@suezuniv.edu.eg (M.I.A.H.); 4 Mechanical Engineering Department, College of Engineering at Al Kharj, Prince Sattam bin Abdulaziz University, Al Kharj 11942, Saudi Arabia; 5 Department of Mechanical Engineering, Faculty of Engineering and Science, Universitas Nasional, Jakarta 12520, Indonesia; fhlatief@civitas.unas.ac.id; 6 IUT de Tremblay, Université Paris 8, 93290 Tremblay-en-France, France; akrum.abdul-latif@univ-paris8.fr

**Keywords:** additive friction stir deposition, solid-state additive manufacturing, AA2011-T6 aluminum alloy, spindle rotation speed, feed rate, microstructure, mechanical properties

## Abstract

The current work investigates the possibility of fabricating additive manufacturing products in solid-state form, from AA2011-T6 of 40 mm diameter rods as a feedstock, using an additive friction stir deposition (A-FSD) technique. The use of large diameter feedstocks, especially high-strength aluminum alloys (2XXX series), is a challenge, as it necessitates high power and the critical selection of the optimal A-FSD parameters, such as feed rate and spindle rotation speed. The study included applying a wide range of spindle rotation speeds, ranging from 400 to 1200 rpm, at three levels of feeding rates of 1, 3, and 5 mm/min. The AA2011-T6 friction stir deposited parts (FSDPs) were visually evaluated. This was followed by an examination of macrostructures through the thickness of the fabricated specimens. The characterization of microstructures was also carried out using optical microscopy and a scanning electron microscope equipped with advanced EDS analysis. Furthermore, the mechanical properties in terms of hardness and compressive strength of the AA2011-T6 base material (BM) and deposited materials were evaluated. Sound, additively manufactured products were successfully fabricated from 40 mm diameter AA2011-T6 feedstocks using the suggested deposition variables of 600 and 800 rpm spindle speeds and feeding rates of 1, 3, and 5 mm/min. The results indicated that the spindle speed and feeding rate govern the quality of the FSDPs. Furthermore, the axial load during the A-FSD process increased with increasing these parameters. In comparison to the AA2011-T6 BM, the additively deposited materials showed a refined grain structure and uniform dispersion of the fragment precipitates in their continuous multi-layers. The reduction ratio in grain size attains 71.56%, 76%, and 81.31% for the FSDPs processed at 800 rpm spindle speed and feeding rates of 1, 3 and 5 mm/min, respectively, compared to the grain size of BM. The Al_2_Cu and Al_7_Cu_2_Fe intermetallics are detected in the AA2011-T6 BM, and their deposited parts are in different shapes of spherical, almost spherical, irregular, and rod-like shapes. The compressive strength and hardness of the deposited parts increased with increasing spindle speed and feeding speeds. At a spindle speed of 800 rpm and a 5 mm/min feeding rate, the higher hardness and compressive strength gained were 85% and 93%, respectively, from that of the AA2011-T6 feedstock.

## 1. Introduction

As metal product manufacturing costs have increased, sustainable manufacturing techniques for producing complex shapes with high strength-to-weight ratios, that produce limited waste, have emerged [1,2]. In recent years, additive manufacturing (AM) techniques have been established in many areas using 3D printing parts fabricated from metals and alloys [3,4] and other materials [5,6,7] built up in layers until they have reached their finished part shape. 

AM technology has been cutting-edge for the past 30 years [8,9,10,11]. There are numerous techniques for additive manufacturing. Firstly, fusion-based additive manufacturing (F-BAM) [12,13,14,15] necessitates the application of a concentrated, high-energy source to a wire [16,17]. These techniques generate large heat gradients, which pose many issues for alloys that are generally difficult or unwieldy to work with, such as aluminum or magnesium alloys. With commercially accessible materials, these fusion methods impose technical limitations in creating as-deposited defect-free aluminum components [12,18]. Secondly, solid-state additive manufacturing based on friction stir technology principles [19,20], known as friction stir additive manufacturing (FSAM), can produce high-quality, layer-by-layer material products [21,22]. It demonstrates increased production speed, mechanical properties, and reduced material and energy waste compared to the F-BAM methods [19]. 

The FSAM process aims to produce a higher-quality product with superior dimensional accuracy and material properties [23]. The primary benefit of the FSAM process is that it eliminates all melting and solidification issues. The FSAM is divided into many processes: additive friction stir surfacing (AFSS) [24]; friction stir welding additive manufacturing (FSW-AM) [25]; and additive friction stir deposition (A-FSD) [26,27]. The A-FSD process is a thermomechanical process based on friction stirring (FS) principles similar to friction stir welding (FSW) [28,29,30] and friction stir processing (FSP) [31,32,33,34] techniques. 

The mechanism of A-FSD consists of a rotating consumable feedstock and a fixed substrate plate. This feedstock rod, while rotating, approaches the fixed plate at a specific feeding speed that generates frictional heat between the rotating feedstock rod and the fixed plate at the contact surface. The generated frictional heat softens and plastically deforms the feedstock rod material, depositing it on the substrate. The deposited part is built layer by layer to produce the desired height [35,36]. In recent years, there has been an increase in demand for applying the A-FSD technology in various applications, such as designing stringer configurations and fabricating stiffeners for the aerospace sector, pressure vessels, and other structural systems [1,37]. 

Dilip et al. [38] used the A-FSD process to build friction stir deposited parts (FSDPs) utilizing 20 mm diameter rods of AA2014-T6. Samples were constructed layer-by-layer, with well-bonded layers, fine grains, and refined second-phase particles. 

Galvis et al. [39] used the A-FSD process, with AA6351-T6 alloy consumable feed-rods of 19 mm in diameter, and reported that the microstructure of the deposited material revealed a fine-grain structure, and the hardness values of the deposited layers showed a reduction in hardness of 25.4% compared to the initial material. 

Elfishawy et al. [26] produced FSDPs using the A-FSD process through the use of Al-Si consumable feedstock rods of 20 mm diameter against the AA5083 substrate plate, using different feeding speeds of 3, 4, and 5 mm/min at a constant spindle speed of 1200 rpm. They reported that the microstructure characteristics included dynamically recrystallized, ultra-fine grains with very fine, well-distributed precipitates. However, the hardness decreased when compared to the base material. 

Alzahrani et al. [40] studied the possibility of the continuous multi-layer deposition of as-cast A356 aluminum alloy via the A-FSD. Their results showed the production of FSDPs with a sound structure and refined grain morphology compared to the as-cast material. The FSDPs showed higher hardness than the as-cast alloy. 

Ahmed et al. [41] studied the effect of A-FSD feeding speeds, and two temper conditions of starting material on the FSDPs produced using AA2011 consumable feedstock rods against an AA5083 substrate plate. The microstructures of the produced FSDPs attained equiaxed refined grains compared to the coarse grain of the AA2011 initial rod material. And the Al_2_Cu and Al_7_Cu_2_Fe intermetallics were detected in the AA2011 alloy and the hardness increased when the AA2011 in the O temper condition was used. 

Dilip et al. [42] produced a 20 mm diameter with a 50 mm height of FSDPs with more than 30 layers by A-FSD using AISI 304 austenitic stainless-steel consumable feedstock rod against a mild-steel substrate plate. The produced FSDPs showed dynamic recrystallization with a fine-grained structure compared to the initial rod material and good metallurgical bonding between the deposited layers. 

Based on the available literature data in the field of additive manufacturing based on solid state friction stir deposition, the current work is seen as the first attempt to fill a gap in the literature about how to quantify the integrated process-microstructure-property linkages of the one-step continuous multilayer-deposited materials of the AA2011-T6 consumable feedstock rod above 20 mm diameter on the AA6082 substrate using the A-FSD method. Thus, this work aims to utilize and optimize the A-FSD process variables to produce FSDPs via a consumable feedstock rod of 40 mm diameter. This large diameter (40 mm) is a serious challenge; it needs high power and the critical selection of optimum conditions, such as the feed rate and machine spindle speed, while controlling the axial downward force. Thus, this investigation applied the A-FSD using three levels of feeding speeds of 1, 3, and 5 mm/min, associated with a wide range of spindle speeds, ranging from 400 to 1200 rpm, to optimize the process parameters. In addition, the axial load during the A-FSD process was recorded to study the resistance of the large diameter consumable feedstock AA2011-T6 rod material during the deposition process. The produced materials were visually and microscopically evaluated. The microstructure features and the formed intermetallics were analyzed via an optical microscope (OM) and scanning electron microscope (SEM), respectively. Furthermore, the mechanical properties, in terms of hardness and compressive strength, were examined and discussed in light of the obtained microstructure.

## 2. Materials and Methods

**Figure 1:**
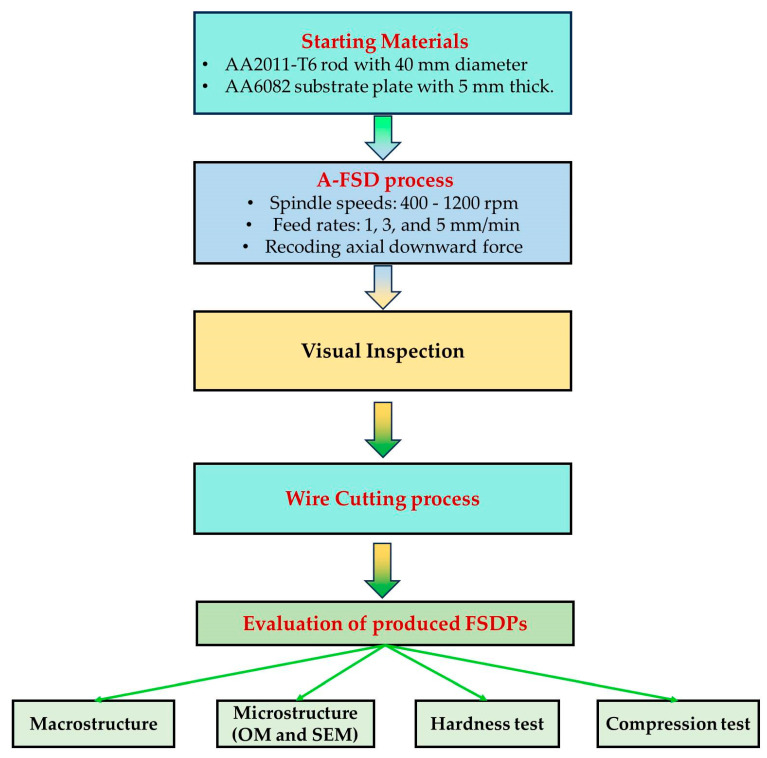
A flowchart summarizes the experimental procedure.

The A-FSD experiments were conducted on the FSW/FSP machine (Model: EG-FSW-M1, Suez University, Suez, Egypt) [43] using aluminum alloy AA2011-T6 as consumable feedstock rods, having 40 mm diameter and 160 mm length, with an effective length of 110 mm. Meanwhile, AA6082 aluminum alloy sheets, with dimensions of 8 mm thickness, 14 mm width, and 20 mm length, were used as substrate plates. Figure 1: A flowchart summarizes the materials and methodology of the current study. 

The chemical composition of the AA2011-T6 rod and AA6082 substrate materials was carried out using Foundry-Master pro (Oxford Instruments, Abingdon, UK) and listed in Table 1. 

**Figure 2:**
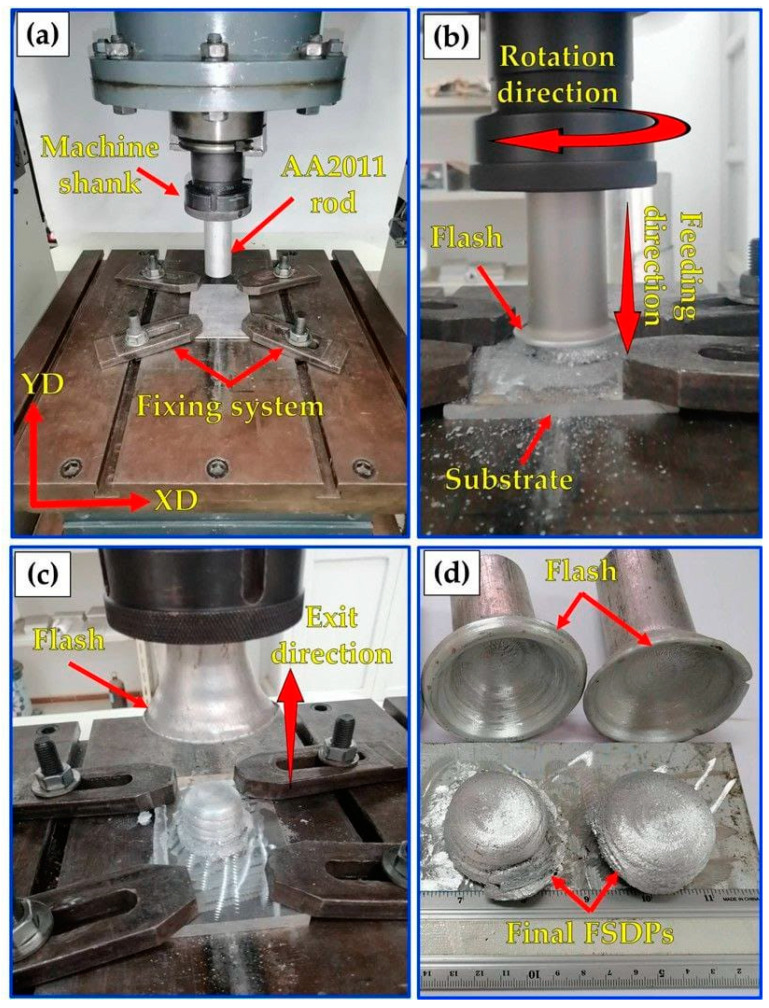
Photo images of the A-FSD process of AA2011-T6 to produce the FSDPs: (**a**) set up of the A-FSD process, (**b**) consumable rod feeding process, (**c**) end of the deposition process, and (**d**) the final FSDPs and their remaining rods.

**Figure 3:**
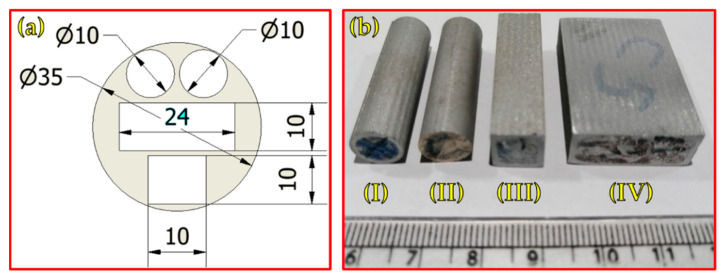
(**a**) Sketch of the dimensions of test specimens and (**b**) photo image of test specimens extracted from the FSDPs: specimens of (**I**,**II**) compression test, (**III**) microstructure test, and (**IV**) macrostructure and hardness tests.

Before starting the A-FSD process, the AA2011-T6 consumable rod was fixed in the machine shank, and the AA6082 plate was fixed on the machine table (Figure 2a). Once the process began, the consumable rod tip was moved down to meet the substrate surface. After that, the consumable rod was rotated at different rotational speeds (400, 600, 800, 1000, and 1200 rpm) under varying feed rates (1, 3, and 5 mm/min). For all the deposited products, the rotating direction of spindle speed was clockwise, and the direction of feeding rate was in the vertical direction (YD), as illustrated in Figure 2b. The rotating rod plasticizes during the rubbing process because of the heat generated from the stirring action between the rotating rod and substrate plate. This supports the rod’s softening, which aids in the material’s deposition from the rod to the substrate, resulting in the formation of the first deposited layer. As the process continued, additional layers were deposited to produce friction stir deposited parts (FSDPS), as shown in Figure 2c,d. For different evaluation tests, the produced FSDPs were cut vertically by a wire cut machine (DK77 High-Speed EDM wire cutting machine, Jiangsu, China) in the deposit direction (YD), and the longitudinal sections are shown in Figure 3a,b. After that, the extracted samples from the FSDPs were machined to correspond with the applied ASTM standards, as listed in Table 2.

The test specimens were prepared according to the standard ASTM E92 metallographic grinding procedures and mechanical polishing for macrostructure and microstructure evaluation. And the test specimens were etched with Keller’s regent solution (3 mL hydrofluoric acid, 6 mL nitric acid, and 90 mL distilled water) for 1 min and dried with forced air after washing with distilled water. The optical microscopic investigation and grain size analysis were conducted using an optical microscope (Olympus, model BX41M-LED, Tokyo, Japan). The SEM microstructure and EDS analysis for the AA2011 initial material and their FSDPs were carried out using the QUANTA FEG 250 (FEI Company, Hillsboro, OR, USA). 

**Figure 4:**
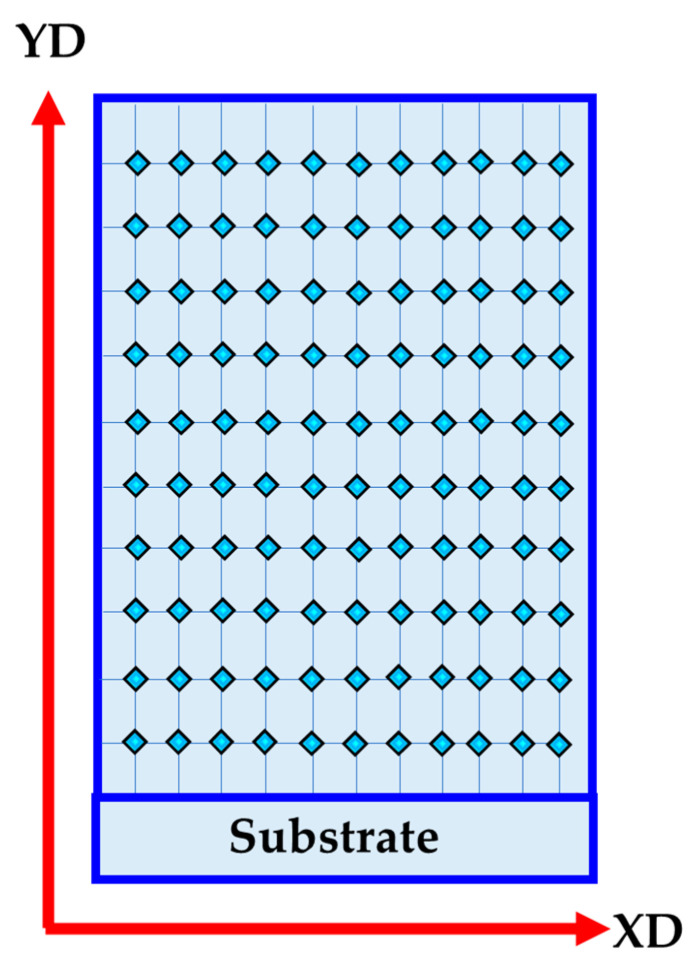
Schematic drawing of hardness map measurements of the produced FSDPs.

Hardness tests were conducted on the AA2011-T6 BM, and the produced FSDPs using a Vickers hardness machine (HWDV-75, TTS Unlimited, Osaka, Japan) carrying a 200 gm for 15 s dwell time, according to the ASTM E92. The test specimens for hardness measurement were taken from the surface parallel to the deposition direction (YD) and at least 122 readings (11 lines in YD and 11 lines in XD) were taken on each specimen, as shown in Figure 4. 

The compression test was also performed to evaluate the strength of the FSDPs using cylindrical samples prepared according to ASTM E9, and the compression force was applied parallel to the YD. The cross-section area of each specimen was 78.54 mm^2^. The test was conducted on a universal testing machine (Instron 4208, 30-ton capacity, Norwood, MA, USA) at room temperature with an applied crosshead speed of 0.05 mm/s.

## 3. Results and Discussions 

### 3.1. Process Observations and Optimization

**Figure 5:**
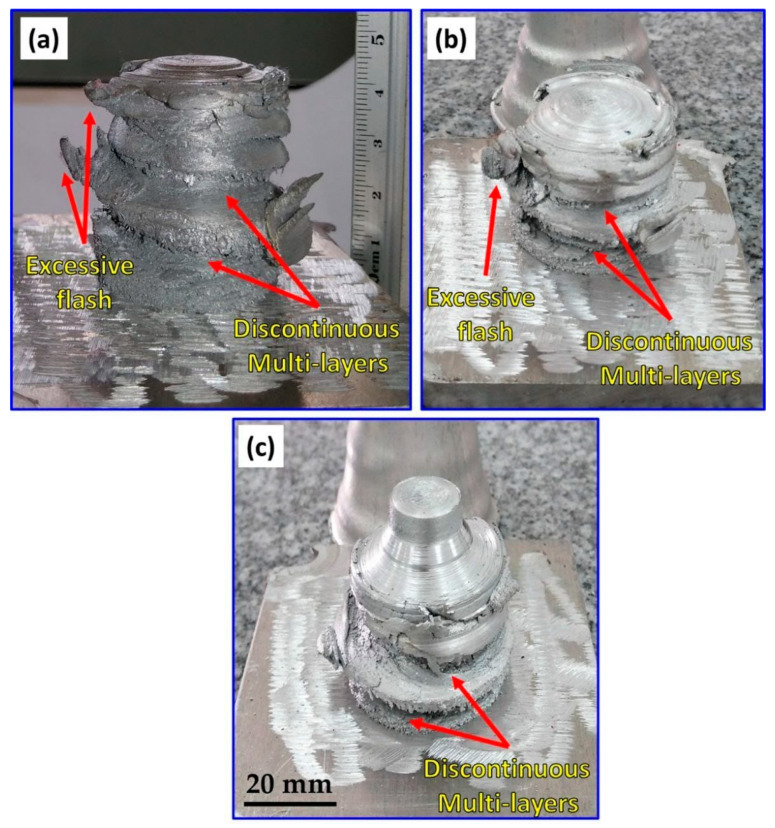
Photographs of AA2011 FSDPs fabricated using a constant feeding speed of 1 mm/min and different rotation rates of (**a**) 1200 rpm, (**b**) 1000 rpm, and (**c**) 400 rpm.

The main objective of this work was to examine the feasibility of friction stir deposition using a large diameter feedstock of 40 mm diameter at a wide range of rotation rates and feeding speeds. Initially, the A-FSD process was conducted at low (400 rpm) and high (1000 and 1200 rpm) rotation rates at a low feeding speed of 1 mm/min. The images of the FSDPs and their remaining feedstock rods after the A-FSD, at a constant feeding speed of 1 mm/min and different rotation rates of 1200, 1000, and 400 rpm, are presented in Figure 5a–c. It can be observed that the FSDPs are not regular in shape at 1200 rpm (Figure 5a), 1000 rpm (Figure 5b), and 400 rpm (Figure 5c) using the low feeding speed of 1 mm/min. This can be attributed to the excessive heat generated at the high rotation rates (1200 and 1000 rpm), resulting in a softened material state that does not allow continuous and regular material deposition. While the low rotation rate (400 rpm) can be attributed to the insufficient heat input that does not allow full consolidation between layers due to high resistance upon deposition. Based on the obtained FSDPs morphologies and surface appearance, it can be concluded that the very high and very low rotation rates are not suitable for manufacturing sound and regular FSDP from the AA2011-T6 feedstock of 40 mm diameter. 

**Figure 6:**
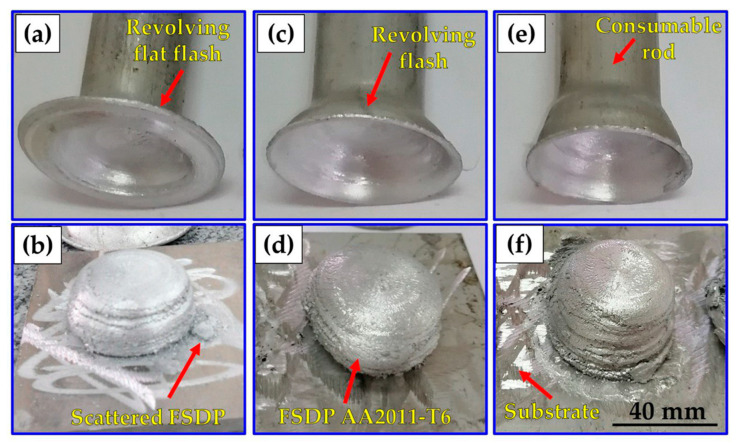
Photographs of the AA2011-T6 FSDPs deposited using different feed rates of (**a**,**b**) 1 mm/min, (**c**,**d**) 3 mm/min, and (**e**,**f**) 5 mm/min at 600 rpm spindle speed.

Figure 6 shows the photo-images of the AA2011 FSDPs and their remaining feedstock rods after the A-FSD using a constant rotation rate of 600 rpm and different feeding speeds of 1 mm/min (Figure 6a,b), 3 mm/min (Figure 6c,d), and 5 mm/min (Figure 6e,f). It can be observed that the FSDPs are regular in shape and continuously formed to the required height. There is a noticeable increase in the height of the fabricated parts by increasing the feeding speed, which increased from 25 mm to 31 mm. Also, another important feature can be noted in the remaining feedstock rods, where quite a large and flat cup is formed at the feeding speed of 1 mm/min. This cup gets smaller and circular in shape as the feeding speed increases to 3 and 5 mm/min. It should be mentioned that this cup formation reflects the inhomogeneous deposition of the material as the cup affects the shape of the deposited part and makes it look like a dome in shape. This cup feature in the feedstock might be formed due to the heat treatment condition (T6) of the rod material and the effect of surface air cooling during the process that affect the temperature gradient from the inside to the outside of the feedstock rod. The surface cooling can make the outer surface more cold than the inside of the rod, and this makes it resist the deposition and remain as a surface flash surrounding the deposited part, which is deformed and shaped according to the feeding speed into a more flat or circular cup. This cup feature affects the surface morphology and probably the microstructure of the deposited material due to its friction with the deposited part during the A-FSD process, which produces a rougher surface and might affect the dissolution or coarsening of the second-phase precipitates.

**Figure 7:**
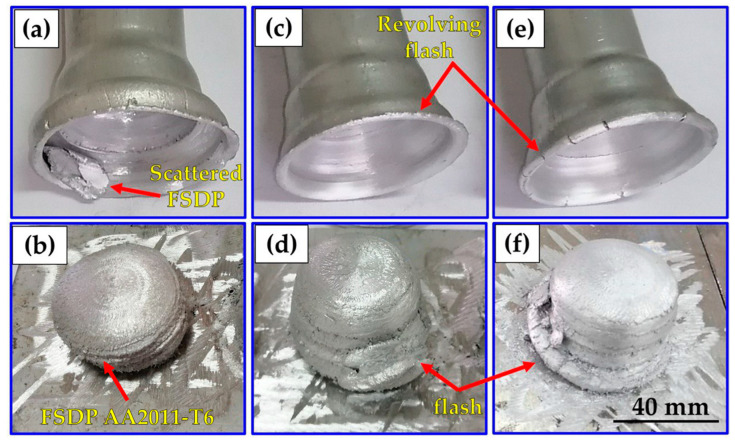
Photographs of the AA2011-T6 FSDPs produced using different feed rates (**a**,**b**) 1 mm/min, (**c**,**d**) 3 mm/min, and (**e**,**f**) 5 mm/min at 800 rpm spindle speed.

Figure 7 shows the photo-images of the AA2011 FSDPs and their remaining feedstock rods after the A-FSD using a constant rotation rate of 800 rpm and different feeding speeds of 1 mm/min (Figure 7a,b), 3 mm/min (Figure 7c,d), and 5 mm/min (Figure 7e,f). It can be observed that the FSDPs are more regular in morphology with homogenous diameter through the whole thickness. The cup feature observed in the remaining feedstock rods looks similar at the low and high feeding speeds with a two-step circular cup, and some cracks can be noted at the high feeding speed. 

### 3.2. Friction Stir Deposition Load

Many parameters control the material deposition behavior in the A-FSD process [43,44]. The most important of these is the axial load. However, few studies have been carried out to study and predict the load during the FSW or FSP process, which can be used to optimize the load during the friction stir deposition process [45,46]. The axial load recorded by the FSW/FSP machine during the A-FSD process is recorded and can be utilized as an indicator for the resistance of the consumable feedstock rod material to deposit on the substrate plate. 

**Figure 8:**
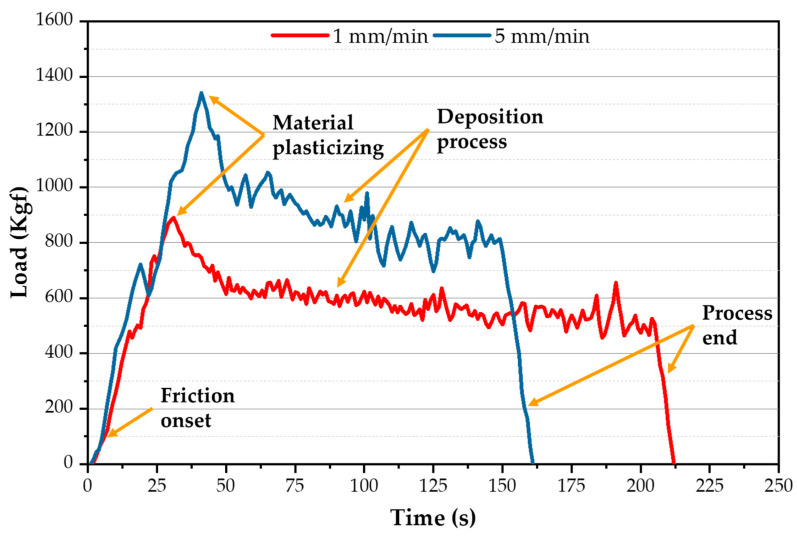
The recorded axial load during the A-FSD process using different feed rates of 1 and 5 mm/min at a spindle speed of 600 rpm.

Figure 8 presents the recorded load values during the A-FSD of AA2011-T6 at feed rates of 1 and 5 mm/min and a spindle speed of 600 rpm. The different regions of load curves obtained during the A-FSD process are consumable feed-rod (AA2011-T6) friction onset, material plasticizing, deposition process, and process end. In the first region, friction onset occurred, when the AA2011-T6 rotating consumable feed rod touched the surface of the AA6082-T6 substrate plate, the recorded load abruptly increased due to mutual friction between the rotating rod and the fixed substrate to accomplish friction onset. In the second region, the material plasticizing region, the recorded load sharply increased to reach its maximum value for all the applied parameters. The continuous feeding rate and stirring action between the rod and the substrate generated sufficient frictional heat to plasticize the rod material and produce the first deposition layer on the AA6082-T6 substrate. In the third region, the deposition process builds FSDPs in continuous multi-layers from down to up with nearly stable load values. The resistance of the materials to deposit depends on the rod material type and the applied process parameters. In the last region the deposition load decreases sharply at the end of the process because the rotating rod leaves upward after finishing the deposition process. Figure 8 shows that the A-FSD time to produce AA2011-T6 FSDPs increases with a decrease in the feed rate from 5 to 1 mm/min. 

**Figure 9:**
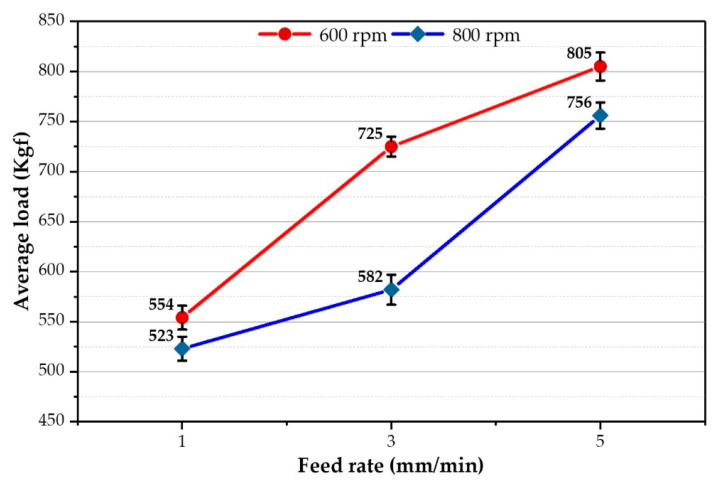
The average axial load of the A-FSD to produce AA2011-T6 FSDPs using rotational speeds of 600 and 800 rpm at feeding speeds of 1, 3, and 5 mm/min.

The average axial load values during the deposition process stage were measured for the spindle speeds of 600 and 800 rpm as a function of feeding speeds of 1, 3, and 5 mm/min (Figure 9). As the feeding speed increases, the average axial load values increase for the two applied spindle speeds of 600 and 800 rpm. And, with decreasing the spindle speed, the heat input decreases. Thus, the load values increase due to the difficulty of material flow [47,48]. Moreover, the material flow with a high spindle speed of 800 rpm and a high 5 mm/min feed rate becomes easier during the FSD process than with applying other parameters. On the other hand, the other parameter conditions provide more resistance to material flow at the lower spindle speeds (600 rpm) and feeding speed (1 mm/min) due to the colder material.

### 3.3. Macrostructure and Microstructure Investigations

**Figure 10:**
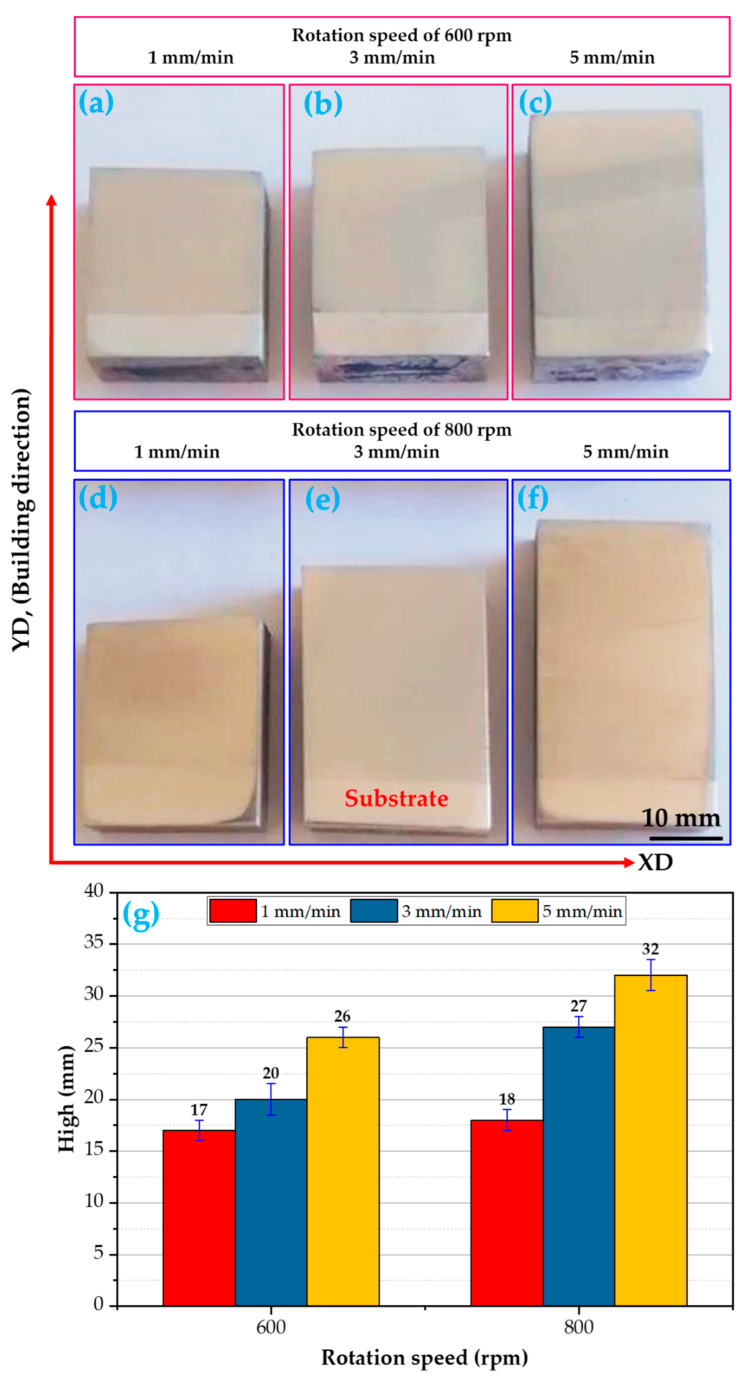
Representation of the macrostructures of AA2011-T6 FSDPs produced using different feeding rats and spindle speeds of 600 rpm (**a**–**c**) and 800 rpm (**d**–**f**). The height of AA2011-T6 FSDPs against the rotational speeds 600 and 800 rpm deposited at different feeding speeds of 1, 3, and 5 mm/min is shown in (**g**).

To understand the A-FSD process, the consumable rod material is under the influence of an applied pressure force and friction at the same time with the substrate plate, which generates an amount of heat input. When adjusting the procedure to conditions that are perfectly suitable for attaining the softening stage of the rod material, a metallurgical bond will arise between the rod and the substrate, and then the layers will successively deposit from bottom to top to create continuous multi-layers, forming a build. The process parameters associated with friction surfacing are the feeding rate, rod rotation speed, axial pressure force, and rod diameter. The macrostructure of the AA2011-T6 FSDPs produced at the two rotational speeds of 600 and 800 rpm and various feeding speeds of 1, 3, and 5 mm/min are shown in Figure 10. It can be noted that the macrostructure of the AA2011-T6 FSDPs shows a solid continuous structure without any bonding discontinuities at the interfaces between the deposited layers. In addition, the visual inspection of the deposited layers revealed almost no accumulated flash. 

Figure 10g illustrates the effect of spindle speeds (600 and 800 rpm) and feeding speeds (1, 2, and 3 mm/min) on the height of the AA2011-T6 FSDPs fabricated using the applied A-FSD technique. It was found that the height of the FSDPs increases with increasing feeding speeds from 1 mm/min to 5 mm/min for both the applied spindle speeds (the rotating speed of the consumable rod). The plasticity of AA2011-T6 during the A-FSD process is controlled by the generated heat input in the stirring area throughout the manufacturing process. The A-FSD heat input during the deposition process is directly related to the feeding speed and the spindle speed of the consumable feed rod. 

**Figure 11:**
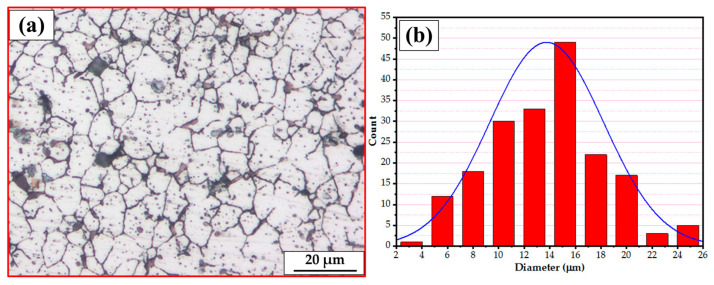
(**a**) The OM microstructures and (**b**) the grain size analysis of the initial rod material c AA2011-T6.

The Figure 11 illustrates the microstructure of the AA2011-T6 initial rod material (Figure 11a) and its related grain size histogram (Figure 11b). The grain size analysis shows that the grain size ranges from 8.62 µm to 25.32 µm, with an average value of 13.75 µm.

Friction-based processes (FSW, FSP, and A-FSD) contribute to increasing the temperature of the processed material in the stirring zone to temperatures that may reach, under the control of the processing parameters, between 60% and 80% of the material’s melting point, which is high enough for recrystallization during the intensive plastic deformation [49,50,51]. The optical microscopy investigation was carried out on long transverse cross-sections of the FSDPs.

**Figure 12:**
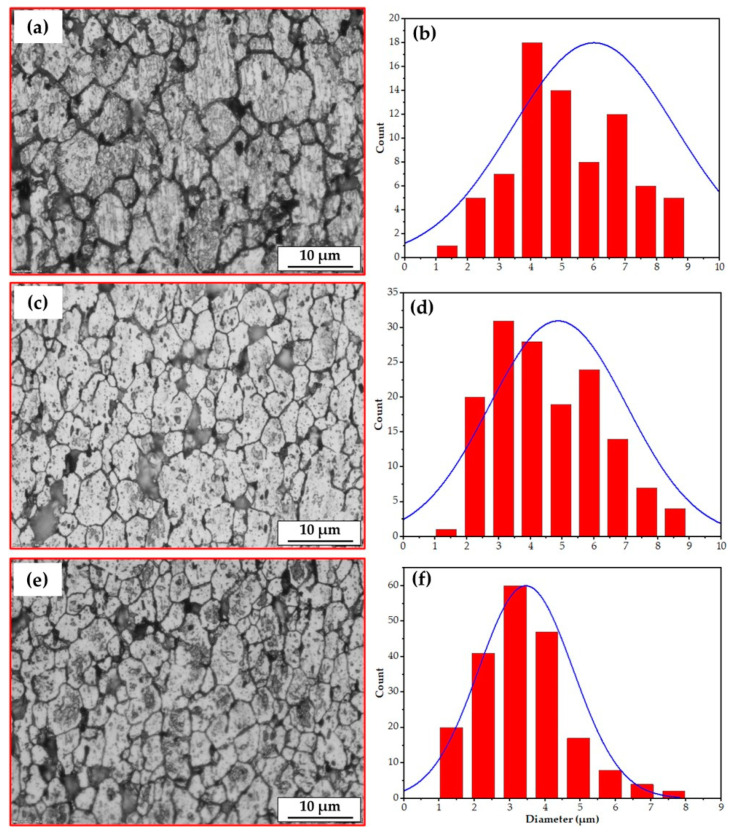
The microstructures and grain size analysis of the AA2011-T6-deposited materials at different feeding speeds of (**a**,**b**) 1 mm/min, (**c**,**d**) 3 mm/min, and (**e**,**f**) 5 mm/min, and at a constant spindle speed of 600 rpm.

**Figure 13:**
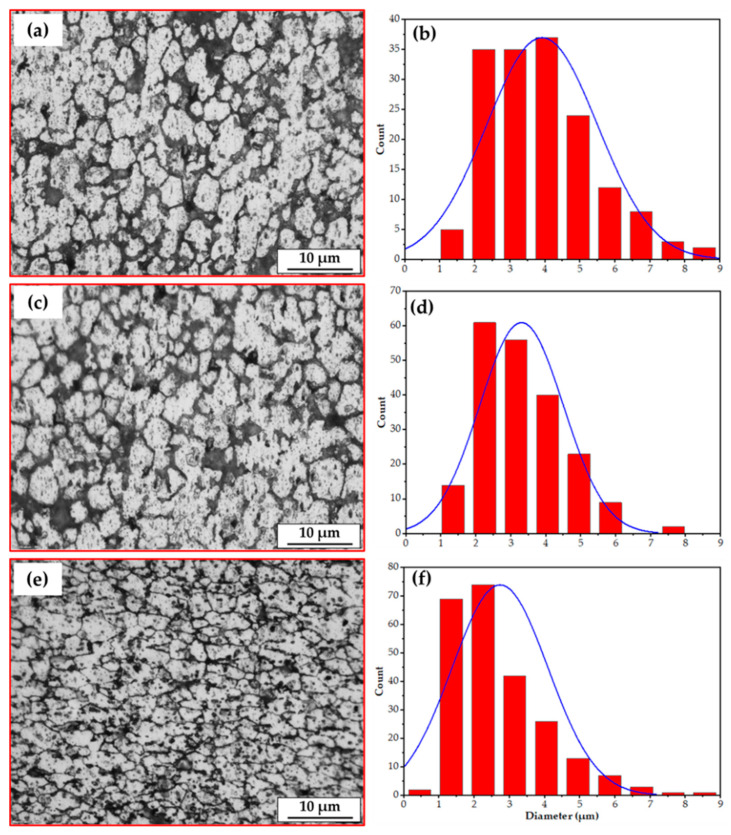
The microstructures and grain size analysis of the AA2011-T6-deposited materials at different feeding speeds of (**a**,**b**) 1 mm/min, (**c**,**d**) 3 mm/min, and (**e**,**f**) 5 mm/min, and at a constant spindle speed of 800 rpm.

Figure 12 and Figure 13 represent the microstructures and grain size analysis of the AA2011-T6 FSDPs produced using 600 and 800 rpm spindle speeds at different feeding speeds of 1, 3, and 5 mm/min. It can be remarked that the AA2011-T6 coarse grain structure and precipitates of the initial consumable feed rod (Figure 11a) are refined with the applied A-FSD parameters. The A-FSD process produced significant grain refining with average grain sizes of 6.00, 4.86, and 3,45 µm at feeding speeds of 1, 3, and 5 mm/min, respectively, at a spindle speed of 600 rpm/min. The reduction percentage in grain size of was 56.36%, 64.65%, and 74.90%, respectively, compared to the grain size of the AA2011-T6 BM, as shown in Figure 12. In Figure 13, the average measured grain sizes of AA2011-T6 FSDPs, produced at a spindle speed of 800, were 3.91, 3.30, and 2.57 µm at feeding speeds of 1, 3, and 5 mm/min, respectively. For the deposited materials, it can be said that the A-FSD reduced the grain size to 71.56%, 76%, and 81.31% of the BM at feeding speeds of 1, 3, and 5 mm/min, respectively. Similar results of fine grains and refined precipitates are noted by Dilip et al. [38] for the multi-layered A-FSD of the Al-Cu-Mg-Si alloy system. Consequently, the deposited materials undergo continuous dynamic recrystallization and develop fine equiaxed grains and refined second-phase particles [52]. Furthermore, Gandra et al. [53] noted a marked reduction in the grain size and intermetallics after the A-FSD surfacing processing of AA2014 alloy material. A homogenous fine equiaxed grain structure has been detected at all the applied conditions due to the dynamic recrystallization during the additive-manufacturing-based friction techniques [54].

**Figure 14:**
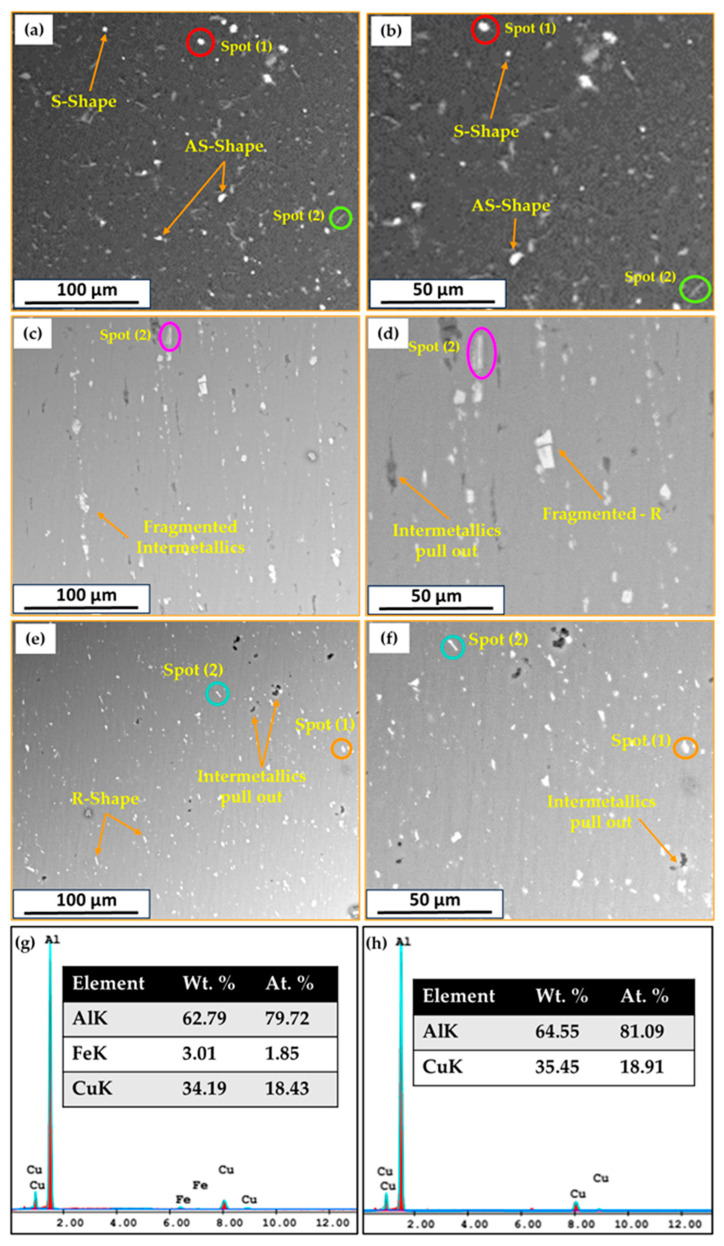
Low- and high-magnification SEM images of (**a**,**b**) AA2011-T6 BM and the produced AA2011-T6 FSDPs at 800 rpm spindle speed and feeding speeds of (**c**,**d**) 1 mm/min and (**e**,**f**) 5 mm/min. (**g**,**h**) are the EDS analysis of spot 1 and spot 2, respectively.

SEM equipped with an EDS analysis technique was applied to investigate the presence of possible intermetallics in the AA2011-T6 initial rod materials and the yielded FSDPs. Table 3 summarizes the detected intermetallics and their morphology for the AA211-T6 consumable rod material and the FSDPs produced at a spindle speed of 800 rpm with applying feeding speeds of 1 and 5 mm/min. According to the SEM-EDS analysis of the used AA2011 alloy, only two intermetallics of Al_2_Cu and Al_7_Cu_2_Fe were detected [43,55]. The Al_2_Cu intermetallic is found in three shapes—spherical (S), almost spherical (A–S), and irregular (I)—in the AA2011-T6 BM and in their FSDPs, while the Al_7_Cu_2_Fe intermetallic was detected in a rod-like (R) shape, as given in Table 3 and shown in Figure 14a–f. The nominal compositions of the two intermetallics of Al_2_Cu (Spot 1 in Figure 14b,c,f) and Al_7_Cu_2_Fe (Spot 2 in Figure 14b,d,f) are represented in Figure 14g,h, respectively. Moreover, it can be noted that these intermetallics in the AA2011-T6 BM (Figure 14a,b) were fragmented and redispersed in the AA2011-T6 FSDPs (Figure 14c–f). This fragmentation of the intermetallics is due to the stirring action during the A-FSD of AA2011-T6 to produce FSDPs; these phenomena are remarked upon and reported in other works [43]. Also, the pullout of the intermetallics in the AA2011-T6 FSDPs after the A-FSD process is observed in a few locations as a result of the grinding process for SEM examination, as shown in Figure 14e–f. The pullout phenomenon is likely due to the weak interface bond between the intermetallics and the aluminum matrix after A-FSD [43].

### 3.4. Mechanical Properties

**Figure 15:**
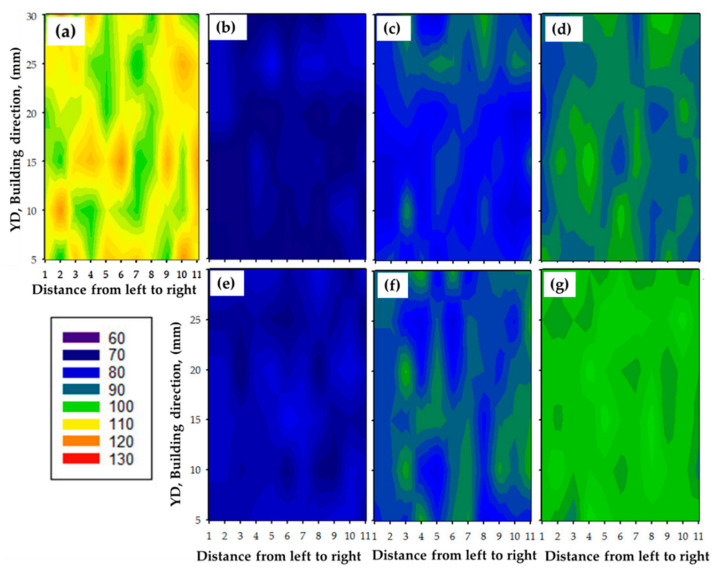
Hardness contour maps of (**a**) AA2011-T6 BM. (**b**–**d**) the FSDPs produced at spindle speed 600 rpm with applying 1, 3, and 5 mm/min feed rates, respectively, while (**e**–**g**) show the FSDPs produced at spindle speed 800 rpm with applying 1, 3, and 5 mm/min feed rates, respectively.

One of the most important indications of the microstructure change that occurs during friction stir deposition is hardness. It is considered an indicator of a material’s mechanical properties, and its value is related to chemical composition, deformation processes, and heat treatment programs. Thus, the hardness was measured on the longitudinal cross-sections of all the built materials produced at different deposition conditions, and these values were represented in contour maps to assess the extent of homogeneity of these materials in terms of microstructure, which shows the direct impact on the measured hardness values. Average hardness values were calculated for each production condition and compared to the AA2011-T6 consumable rod. The colored hardness contour maps of Vickers hardness measurements of the AA2011-T6 BM and the deposited materials that were processed at spindle speeds of 600 and 800 rpm and different rod feeding rates of 1, 3, and 5 mm/min are given in Figure 15. These hardness variations, through the cross-sections of the tested samples, indicate the variations of the microstructure features (Figure 11, Figure 12 and Figure 13) with applying the material processing condition. 

It can be noted from Figure 15a that the initial AA2011-T6 has a wide range of hardness measurements, from 106 to 130 HV, with an average hardness value of 117 HV. The high hardness of the AA2011-T6 rod BM is attributed to the precipitation hardening effect [56,57]. The hardness of AA2011-T6 FSDPs, produced at a spindle speed of 600 rpm with applying feeding rates of 1, 3, and 5 mm/min (Figure 15b–d), ranged from 68 to 81, from 78 to 93, and from 84 to 95 HV, respectively. At the same time, the deposited materials at 800 rpm spindle speed follow the same trend in hardness values. As given in Figure 15e–g, a narrow range of hardness measurements is given from 74 to 88, from 78 to 89, and from 87 to 94 HV for the FSDPs produced at feeding rates of 1, 3, and 5 mm/min, respectively, with a spindle speed of 800 rpm. It can be said that, after the FSD process of AA2011-T6, the FSDPs exhibit a more homogenous hardness distribution at the different deposition conditions compared to the initial rod material, indicating an isotropy structure.

**Figure 16:**
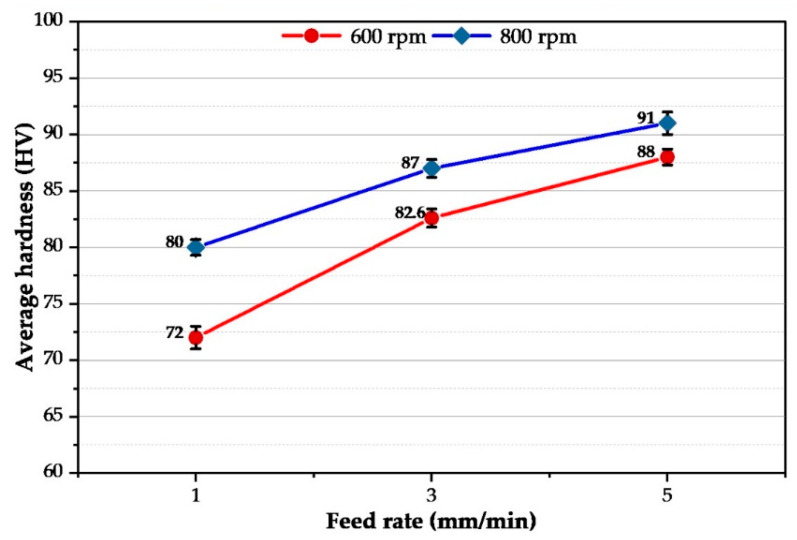
The average hardness of the AA2011-deposited materials as a function of feeding rate (1, 3, and 5 mm/min) at 600 and 800 rpm spindle speed.

The average hardness value was plotted against the consumable feed rate of AA2011-T6 rod material at the two applied rotating speeds of 600 and 800 rpm/min, Figure 16. It can be remarked that, as the feeding rate increases from 1 to 5 mm/min, the hardness value of the FSDPs increases from 80 to 91 HV for the deposited layers at a rotating speed of 800 rpm. The hardness at the lower rotating rod speed of 600 rpm increased the hardness from 72 to 88 HV with raising the consumable feeding rate from 1 to 5 mm/min. Numerous studies [41] have proven that high-strength aluminum alloys of type T6 in the stirred zones, when exposed to friction stir welding or processing, as well as when layered deposition occurs via a solid state stir action (A-FSD), the influence of T6 treatment will be lost in the compensation of forming small grain sizes compared to the parent material, but this decrease in the size of the grain does not contribute to a significant enhancement in hardness as the precipitation hardening For the net measured hardness values for the FSDPs, the most affected factor is the role of precipitation in terms of morphology, dissolution, dispersion, or growth. The deposition conditions of 800 rpm and 5 mm/min attain 85% of the hardness of the AA2011-T6 rod material.

**Figure 17:**
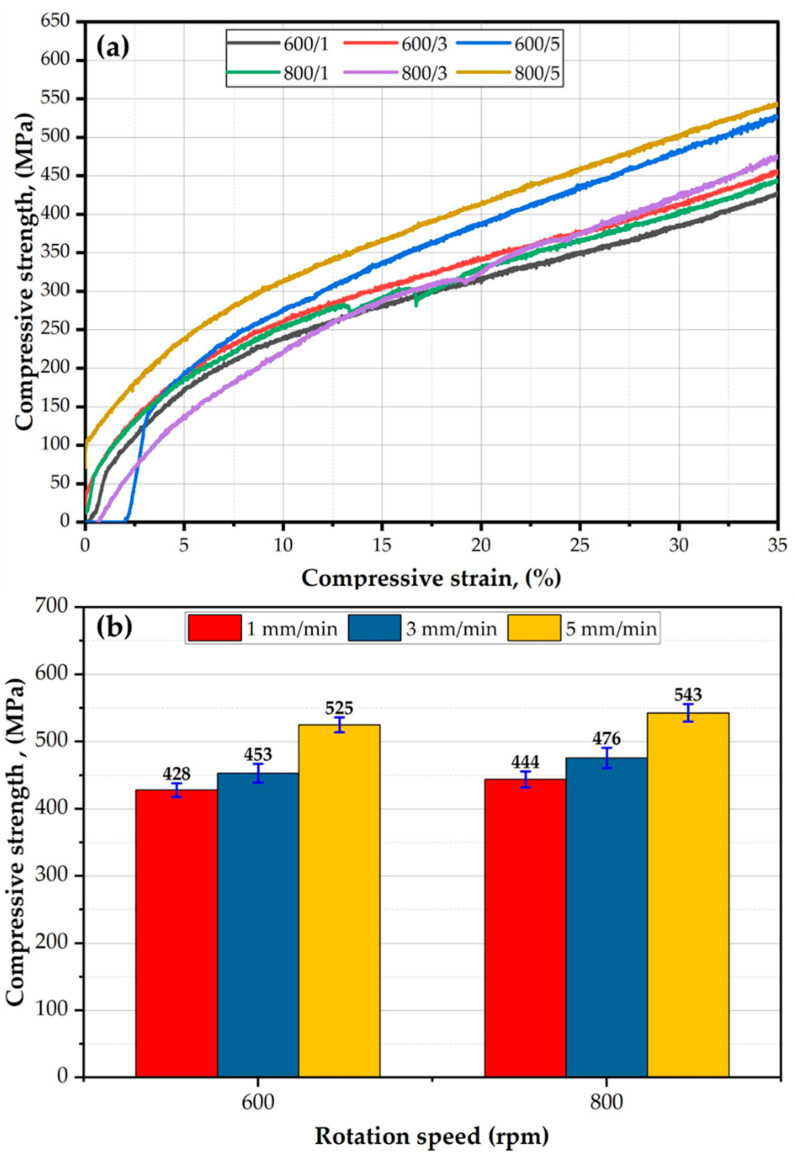
(**a**) Stress–strain compression curves of the AA2011-deposited materials at different deposition conditions, and (**b**) the compressive strength at a strain of 35% of deposited materials as a function of consumable rod rotational speed.

The compression test determines how the materials behave under compressive loads. Figure 17a plotted the compression stress–strain curves, and Figure 17b the compressive strength at 35% strain of the AA2011-T6 FSDPs, deposited at the two rotation speeds of 600 and 800 rpm and feeding rates of 1, 3, and 5 mm/min. It can be seen that all the deposited materials deformed up to 35% strain without any failure. In addition, the compressive strength of the specimen deposited at 800 rpm and 5 feeding rates showed the highest compressive strength behavior during the compression test, whereas the specimen process at the deposition condition of 600 rpm and 1 mm/min gave the lowest compressive strength values (Figure 17a). These results agree well with the hardness results. At 35% strain, the compressive strength of the tested materials increases with increasing deposition rates from 1 to 5 mm/min at the two applied consumable rod AA2011-T6 rotation speeds of 600 and 800 rpm (Figure 17b). And the maximum compressive strength is obtained at the spindle speed of 800 and 5 mm/min feed rate with 93% from the AA2011-T6 BM. Similar findings were reported by El-Sayed Seleman et al. [43] for AA2011-T6/1.5 vol% Al_2_O_3_ rod composite materials deposited at deposition conditions of 800 rpm and 5 mm/min. The deposited layers attained a maximum compressive strength value of 95.5% of the initial material.

## 4. Conclusions

1. The suggested A-FSD process successfully fabricated sound, continuous, multi-layer AA2011-T6 FSDPs using AA2011-T6 large diameter feedstocks of 40 mm without porosity, cavity defects, or interfacial discontinuities between the deposited layers, using spindle rotation speeds of 600 and 800 rpm at feeding rates of 1, 3, and 5 mm/min.
2. The height of the produced AA2011-T6 FSDPs increased with increasing spindle rotation speed and feeding rate. The highest-deposited part of 32 mm was constructed at the 800 rpm spindle speed and 5 mm/min feeding speed.
3. The axial load during the A-FSD of AA2011-T6 increased with increasing spindle speed and feeding rates. The highest axial load of 805 Kgf was detected at the spindle rotation speed of 600 rpm and feeding speed of 5 mm/min
4. The highest hardness and compressive strength values were attained for the deposited part produced at 800 rpm and 5 mm/min, and achieved 85% and 93% of the hardness and compressive strength of the AA2011-T6 initial material, respectively.

## Figures and Tables

**Table 1:** Nominal composition of the AA2011-T6 and AA6082-T6 base materials (wt.%).

	AA2011-T6								
Elements	Cu	Si	Fe	Ti	Bi	Zn	Pb	Ni	Al
wt.%	5.12	0.39	0.70	0.31	0.22	0.24	0.20	0.04	Bal.
	**AA6082-T6**								
Element	Mg	Mn	Cu	Zn	Bi	Si	Fe	Ni	Al
wt.%	0.83	0.57	0.05	0.04	0.03	0.84	0.26	0.01	Bal.

**Table 2:** Dimensions of the machined specimens for different tests.

No.	Specimen Dimensions (mm)	Test
**1**	∅10 × 15	Compression
**2**	10 × 10 × 5	Optical microstructure
**3**	10 × 10 × 5	SEM investigation
**4**	25 × 10 × 32	Hardness and macrostructure

**Table 3:** Chemical composition and morphology of the possible intermetallic phases detected in the FSDPs after the A-FSD process.

No	Intermetallics	Morphology	Abbreviation	Chemical Composition (wt.%)
1	Al_2_Cu	Spherical	S	64.55% Al and 35,45 % Cu (Figure 14h)
		Almost spherical	A–S	
		Irregular	I	
2	Al_7_Cu_2_Fe	Rod-like	R	62.79% Al, 34.19% Cu, and 3.01% Fe (Figure 14g)

## Data Availability

Not applicable.

## References

[1] Mondal M., Das H., Hong S., Jeong B. (2019). CIRP Annals—Manufacturing Technology Local Enhancement of the Material Properties of Aluminium Sheets by a Combination of Additive Manufacturing and Friction Stir Processing. CIRP Ann.-Manuf. Technol..

[2] Ngo T.D., Kashani A., Imbalzano G., Nguyen K.T.Q., Hui D. (2018). Additive Manufacturing (3D Printing): A Review of Materials, Methods, Applications and Challenges. Compos. Part B Eng..

[3] Mishra R.S., Palanivel S. (2017). Building without Melting: A Short Review of Friction-Based Additive Manufacturing Techniques. Int. J. Addit. Subtractive Mater. Manuf..

[4] Padhy G.K., Wu C.S., Gao S. (2017). Friction Stir Based Welding and Processing Technologies—Processes, Parameters, Microstructures and Applications: A Review. J. Mater. Sci. Technol..

[5] Harun W.S.W., Kamariah M.S.I.N., Muhamad N., Ghani S.A.C., Ahmad F., Mohamed Z. (2018). A Review of Powder Additive Manufacturing Processes for Metallic Biomaterials. Powder Technol..

[6] Singh S., Ramakrishna S., Singh R. (2017). Material Issues in Additive Manufacturing: A Review. J. Manuf. Process..

[7] Farzaneh A., Khorasani M., Farabi E., Gibson I., Leary M., Ghasemi A.H., Rolfe B. (2022). Sandwich Structure Printing of Ti-Ni-Ti by Directed Energy Deposition. Virtual Phys. Prototyp..

[8] Nitoi A., Cristea D., Pop M.A., Bedo T., Varga B., Munteanu D. (2019). Aluminum Based Metastable Alloys for Additive Manufacturing. IOP Conf. Ser. Mater. Sci. Eng..

[9] Guofang L. (2021). Research on Structural Design of Aluminum Components for Large Aircraft Based on Additive Manufacturing Technology. J. Phys. Conf. Ser..

[10] Bahrami B., Mehraban M.R., Koloor S.R., Ayatollahi M.R. (2023). Non-Local and Local Criteria Based on the Extended Finite Element Method (XFEM) for Fracture Simulation of Anisotropic 3D-Printed Polymeric Components. Rapid Prototyp. J..

[11] Kumar Srivastava A., Kumar N., Rai Dixit A. (2021). Friction Stir Additive Manufacturing—An Innovative Tool to Enhance Mechanical and Microstructural Properties. Mater. Sci. Eng. B Solid-State Mater. Adv. Technol..

[12] Zhang D., Sun S., Qiu D., Gibson M.A., Dargusch M.S., Brandt M., Qian M., Easton M. (2018). Metal Alloys for Fusion-Based Additive Manufacturing. Adv. Eng. Mater..

[13] Liu G., Xiong J., Tang L. (2020). Microstructure and Mechanical Properties of 2219 Aluminum Alloy Fabricated by Double-Electrode Gas Metal Arc Additive Manufacturing. Addit. Manuf..

[14] Qi Z., Qi B., Cong B., Sun H., Zhao G., Ding J. (2019). Microstructure and Mechanical Properties of Wire + Arc Additively Manufactured 2024 Aluminum Alloy Components: As-Deposited and Post Heat-Treated. J. Manuf. Process..

[15] Oropeza D., Hofmann D.C., Williams K., Firdosy S., Bordeenithikasem P., Sokoluk M., Liese M., Liu J., Li X. (2020). Welding and Additive Manufacturing with Nanoparticle-Enhanced Aluminum 7075 Wire. J. Alloys Compd..

[16] Herderick E.D. (2015). Progress in Additive Manufacturing. JOM.

[17] Frazier W.E. (2014). Metal Additive Manufacturing: A Review. J. Mater. Eng. Perform..

[18] Mertens A.I., Delahaye J., Lecomte-Beckers J. (2017). Fusion-Based Additive Manufacturing for Processing Aluminum Alloys: State-of-the-Art and Challenges. Adv. Eng. Mater..

[19] Fouly A., Alnaser I.A., Assaifan A.K., Abdo H.S. (2023). Developing PMMA/Coffee Husk Green Composites to Meet the Individual Requirements of People with Disabilities: Hip Spacer Case Study. J. Funct. Biomater..

[20] Gopan V., Leo Dev Wins K., Surendran A. (2021). Innovative Potential of Additive Friction Stir Deposition among Current Laser Based Metal Additive Manufacturing Processes: A Review. CIRP J. Manuf. Sci. Technol..

[21] Perry M.E.J., Griffiths R.J., Garcia D., Sietins J.M., Zhu Y., Yu H.Z. (2020). Morphological and Microstructural Investigation of the Non-Planar Interface Formed in Solid-State Metal Additive Manufacturing by Additive Friction Stir Deposition. Addit. Manuf..

[22] Rivera O.G., Allison P.G., Brewer L.N., Rodriguez O.L., Jordon J.B., Liu T., Whittington W.R., Martens R.L., Mcclelland Z., Mason C.J.T. (2018). Materials Science & Engineering A In Fl Uence of Texture and Grain Re Fi Nement on the Mechanical Behavior of AA2219 Fabricated by High Shear Solid State Material Deposition. Mater. Sci. Eng. A.

[23] Xia M., Nematollahi B., Sanjayan J. (2019). Printability, Accuracy and Strength of Geopolymer Made Using Powder-Based 3D Printing for Construction Applications. Autom. Constr..

[24] Anderson-Wedge K., Avery D.Z., Daniewicz S.R., Sowards J.W., Allison P.G., Jordon J.B., Amaro R.L. (2021). Characterization of the Fatigue Behavior of Additive Friction Stir-Deposition AA2219. Int. J. Fatigue.

[25] Vidakis N., Petousis M., Korlos A., Mountakis N., Kechagias J.D. (2022). Friction Stir Welding Optimization of 3D-Printed Acrylonitrile Butadiene Styrene in Hybrid Additive Manufacturing. Polymers.

[26] Elfishawy E., Ahmed M.M.Z., El-Sayed Seleman M.M. (2020). Additive Manufacturing of Aluminum Using Friction Stir Deposition. TMS 2020 149th Annual Meeting & Exhibition Supplemental Proceedings.

[27] Rivera O.G. (2017). Structure-Property Relationships of Solid State Additive Manufactured Aluminum Alloy 2219 and Inconel 625.

[28] Trueba L., Torres M.A., Johannes L.B., Rybicki D. (2018). Process Optimization in the Self-Reacting Friction Stir Welding of Aluminum 6061-T6. Int. J. Mater. Form..

[29] Longhurst W.R., Cox C.D., Gibson B.T., Cook G.E., Strauss A.M., Wilbur I.C., Osborne B.E. (2017). Development of Friction Stir Welding Technologies for In-Space Manufacturing. Int. J. Adv. Manuf. Technol..

[30] Jandaghi M.R., Pouraliakbar H., Hong S.I., Pavese M. (2020). Grain Boundary Transition Associated Intergranular Failure Analysis at TMAZ/SZ Interface of Dissimilar AA7475-AA2198 Joints by Friction Stir Welding. Mater. Lett..

[31] El-Rayes M.M., El-Danaf E.A. (2012). The Influence of Multi-Pass Friction Stir Processing on the Microstructural and Mechanical Properties of Aluminum Alloy 6082. J. Mater. Process. Technol..

[32] Yang R., Zhang Z., Zhao Y., Chen G., Guo Y., Liu M., Zhang J. (2015). Effect of Multi-Pass Friction Stir Processing on Microstructure and Mechanical Properties of Al3Ti/A356 Composites. Mater. Charact..

[33] Asadi P., Faraji G., Masoumi A., Givi M.K.B. (2011). Experimental Investigation of Magnesium-Base Nanocomposite Produced by Friction Stir Processing: Effects of Particle Types and Number of Friction Stir Processing Passes. Metall. Mater. Trans. A Phys. Metall. Mater. Sci..

[34] Pouraliakbar H., Beygi R., Fallah V., Hosseini Monazzah A., Reza Jandaghi M., Khalaj G., da Silva L.F.M., Pavese M. (2022). Processing of Al-Cu-Mg Alloy by FSSP: Parametric Analysis and the Effect of Cooling Environment on Microstructure Evolution. Mater. Lett..

[35] Priedeman J.L., Phillips B.J., Lopez J.J., Tucker Roper B.E., Hornbuckle B.C., Darling K.A., Jordon J.B., Allison P.G., Thompson G.B. (2020). Microstructure Development in Additive Friction Stir-Deposited Cu. Metals.

[36] Yu H.Z., Jones M.E., Brady G.W., Griffiths R.J., Garcia D., Rauch H.A., Cox C.D., Hardwick N. (2018). Non-Beam-Based Metal Additive Manufacturing Enabled by Additive Friction Stir Deposition. Scr. Mater..

[37] Hartley W.D., Garcia D., Yoder J.K., Poczatek E., Forsmark J.H., Luckey S.G., Dillard D.A., Yu H.Z. (2021). Solid-State Cladding on Thin Automotive Sheet Metals Enabled by Additive Friction Stir Deposition. J. Mater. Process. Technol..

[38] Dilip J.J.S., Ram G.D.J. (2014). Microstructure Evolution in Aluminum Alloy AA 2014 during Multi-Layer Friction Deposition. Mater. Charact..

[39] Carlos J., Henrique P., Oliveira F., Paula J. (2018). De Assessment of Process Parameters by Friction Surfacing on the Double Layer Deposition. Mater. Res..

[40] Alzahrani B., El-Sayed Seleman M.M., Ahmed M.M.Z., Elfishawy E., Ahmed A.M.Z., Touileb K., Jouini N., Habba M.I.A. (2021). The Applicability of Die Cast A356 Alloy to Additive Friction Stir Deposition at Various Feeding Speeds. Materials.

[41] Ahmed M.M.Z., El M.M., Seleman S., Elfishawy E., Alzahrani B., Touileb K., Habba M.I.A. (2021). The Effect of Temper Condition and Feeding Speed on the Additive Manufacturing of AA2011 Parts Using Friction Stir Deposition. Materials.

[42] Dilip J.J.S., Rafi H.K., Ram G.D.J. (2011). A New Additive Manufacturing Process Based on Friction Deposition. Trans. Indian Inst. Met..

[43] El-Sayed Seleman M.M., Ataya S., Ahmed M.M.Z., Hassan A.M.M., Latief F.H., Hajlaoui K., El-Nikhaily A.E., Habba M.I.A. (2022). The Additive Manufacturing of Aluminum Matrix Nano Al_2_O_3_ Composites Produced via Friction Stir Deposition Using Different Initial Material Conditions. Materials.

[44] Ataya S., Ahmed M.M.Z., El-Sayed Seleman M.M., Hajlaoui K., Latief F.H., Soliman A.M., Elshaghoul Y.G.Y., Habba M.I.A. (2022). Effective Range of FSSW Parameters for High Load-Carrying Capacity of Dissimilar Steel A283M-C/Brass CuZn40 Joints. Materials.

[45] Ahmed M.M.Z., Seleman M.M.E.S., Eid R.G., Albaijan I., Touileb K. (2022). The Influence of Tool Pin Geometry and Speed on the Mechanical Properties of the Bobbin Tool Friction Stir Processed AA1050. Materials.

[46] Hammad A.S., Ahmed M.M.Z., Lu H., El-Shabasy A.B., Alzahrani B., El-Sayed Seleman M.M., Zhang Y., El Megharbel A. (2022). An Investigation on Mechanical and Microstructural Evolution of Stationary Shoulder Friction Stir Welded Aluminum Alloy AA7075-T651. Proc. Inst. Mech. Eng. Part C J. Mech. Eng. Sci..

[47] Peel M.J., Steuwer A., Withers P.J., Dickerson T., Shi Q., Shercliff H. (2006). Dissimilar Friction Stir Welds in AA5083-AA6082. Part I: Process Parameter Effects on Thermal History and Weld Properties. Metall. Mater. Trans..

[48] Phillips B.J., Avery D.Z., Liu T., Rodriguez O.L., Mason C.J.T., Jordon J.B., Brewer L.N., Allison P.G. (2019). Microstructure-Deformation Relationship of Additive Friction Stir-Deposition Al–Mg–Si. Materialia.

[49] Ahmed M.M.Z., Seleman M.M.E., Fydrych D., Cam G. (2023). Friction Stir Welding of Aluminum in the Aerospace Industry: The Current Progress and State-of-the-Art Review. Materials.

[50] Fouad D.M., El-Garaihy W.H., Ahmed M.M.Z., El-Sayed Seleman M.M., Salem H.G. (2018). Influence of Multi-Channel Spiral Twist Extrusion (MCSTE) Processing on Structural Evolution, Crystallographic Texture and Mechanical Properties of AA1100. Mater. Sci. Eng. A.

[51] Chandrashekar A., Ajaykumar B.S.S., Reddappa H.N.N. (2018). Effect of Pin Profile and Process Parameters on the Properties of Friction Stir Welded Al-Mg Alloy. Mater. Today Proc..

[52] Ahmed M.M.Z., Essa A.R.S., Ataya S., Seleman M.M.E., El-aty A.A., Alzahrani B., Touileb K., Bakkar A., Ponnore J.J., Mohamed A.Y.A. (2023). Friction Stir Welding of AA5754-H24: Impact of Tool Pin Eccentricity and Welding Speed on Grain Structure, Crystallographic Texture, and Mechanical Properties. Materials.

[53] Gandra J., Krohn H., Miranda R.M., Vilac P., Quintino L., Santos J.F. (2014). Journal of Materials Processing Technology Friction Surfacing—A Review. J. Mater. Process. Technol..

[54] Khodabakhshi F., Gerlich A.P.P. (2018). Potentials and Strategies of Solid-State Additive Friction-Stir Manufacturing Technology: A Critical Review. J. Manuf. Process..

[55] Grażyna M.N., Gancarczyk K., Nowotnik A., Dychtoń K., Boczkal G. (2021). Microstructure and Properties of As-Cast and Heat-Treated 2017a Aluminium Alloy Obtained from Scrap Recycling. Materials.

[56] Liu C., Yi X. (2013). Residual Stress Measurement on AA6061-T6 Aluminum Alloy Friction Stir Butt Welds Using Contour Method. Mater. Des..

[57] Mansourinejad M., Mirzakhani B. (2012). Influence of Sequence of Cold Working and Aging Treatment on Mechanical Behaviour of 6061 Aluminum Alloy. Trans. Nonferrous Met. Soc. China (Engl. Ed.).

